# Diagnosis of children with attention-deficit/hyperactivity disorder (ADHD) comorbid autistic traits (ATs) by applying quantitative magnetic resonance imaging techniques

**DOI:** 10.3389/fpsyt.2022.1038471

**Published:** 2022-11-17

**Authors:** Shilong Tang, Xianfan Liu, Lisha Nie, Zhuo Chen, Qiying Ran, Ling He

**Affiliations:** ^1^Department of Radiology, Children's Hospital of Chongqing Medical University, National Clinical Research Center for Child Health and Disorders, Ministry of Education Key Laboratory of Child Development and Disorders, Chongqing Key Laboratory of Pediatrics, Chongqing, China; ^2^GE Healthcare, MR Research China, Beijing, China

**Keywords:** children, attention-deficit/hyperactivity disorder, magnetic resonance imaging, autistic traits, brain

## Abstract

**Objective:**

To explore the feasibility of applying quantitative magnetic resonance imaging techniques for the diagnosis of children with attention-deficit/hyperactivity disorder (ADHD) comorbid autistic traits (ATs).

**Methods:**

A prospective study was performed by selecting 56 children aged 4–5 years with ADHD-ATs as the study group and 53 sex- and age-matched children with ADHD without ATs as the control group. All children underwent magnetic resonance scans with enhanced T2^*^- weighted magnetic resonance angiography (ESWAN), 3D-PCASL, and 3D-T1 sequences. Iron content and cerebral blood flow parameters were obtained *via* subsequent software processing, and the parameter values in particular brain regions in both groups were compared and analyzed to determine the characteristics of these parameters in children with ADHD-ATs.

**Results:**

Iron content and cerebral blood flow in the frontal lobe, temporal lobe, hippocampus, and caudate nucleus of children with ADHD-ATs were lower than those of children with ADHD without ATs (*p* < 0.05). Iron content and CBF values in the frontal lobe, temporal lobe and caudate nucleus could distinguish children with ADHD-ATs from those without ATs (AUC > 0.5, *p* < 0.05).

**Conclusions:**

Quantitative magnetic resonance techniques could distinguish children with ADHD-ATs.

**Trial registration:**

This study protocol was registered at the Chinese clinical trial registry (ChiCTR2100046616).

## Highlights

- The results of this study indicate that the brain iron content and CBF values of ADHD-ATs is lower than that of ADHD children.
- CBF and QSM values in the frontal lobe, temporal lobe, and caudate nucleus could distinguish children with ADHD-ATS from children with ADHD without ATs.

## Introduction

Attention-deficit hyperactivity disorder (ADHD) is a frequently encountered neurodevelopmental behavioral disorder that mostly affects children and adolescents. Its main clinical features include hyperactivity, impulsivity, and attention deficit, resulting in serious impacts on the normal growth and development of children (1–3).

Children with ADHD usually have additional mental disorders, namely, autism spectrum disorder (ASD) and depression, which can aggravate the condition and impair social function (4, 5). Children with ADHD who attract the most attention because of more serious conditions, also have ASD (6, 7). ASD is also a common neurodevelopmental behavioral disorder, associated with social communication disorders, repetitive stereotyped behaviors and language developmental disorders as its core manifestations, and the key problem is poor social communication (8, 9). If children have both ADHD and ASD, their social function impairment can be substantially increased compared with patients with ADHD alone (10, 11).

Clinicians have introduced different treatment plans for ADHD children with ASD and those without ASD. If there is a missed diagnosis or misdiagnosis, it may lead to further aggravation of the child's condition (12, 13). Currently, the diagnosis of patients with ADHD comorbid autism is mainly based on the clinical symptoms of the patients and various assessment scales, which are highly subjective and result in a missed diagnosis or misdiagnosis (14, 15). In particular, ADHD children who develop only autistic traits (ATs) and do not reach the threshold for a diagnosis of ASD are more likely to be missed or misdiagnosed (16, 17). ATs mainly refer to several apparent defects in social behavior, language communication ability, environmental adaptability and social perception that have not yet met the diagnostic criteria threshold for ASD (18, 19). Relevant data have suggested that executive function impairments in children with ADHD-ATs may be more complex than the pathological mechanism of brain function involved in children with ADHD without ATs (20, 21). Determining how to diagnose ADHD-ATs in a more scientific way is a hotspot and challenge for medical researchers.

As an important micro element in our body, iron is essential for our health. The iron content in the brain is precisely regulated, any anomalous will lead to physical abnormalities, and too much iron element content will lead to apoptosis of nerve cells; On the contrary, too little will lead to low IQ, irritability, poor reaction and other symptoms (17–21); It's same that too much or too little cerebral blood flow will also lead to abnormal development of the brain region, too little blood flow will lead to slow development of the brain, and too much will lead to excessive development of the brain region (22, 23).

In previous studies, some researchers have found that the iron content and cerebral blood flow in some brain regions of children with ASD and ADHD are lower than those of healthy children (8, 24–26), ADHD-ATs children have some characteristics of ADHD and ASD children at the same time, but it is unknown whether the brain iron content or cerebral blood flow will be further reduced, and whether the brain iron content or cerebral blood flow can be used as a diagnostic marker to distinguish ADHD-ATs from ADHD children.

Multimodal magnetic resonance (3D-T1, ESWAN, and 3D-PCASL sequences) scans of the brains of children with ADHD-ATs and children with ADHD without Ats were performed, and the cerebral 3D-T1 structural plot, QSM quantitative plot, and CBF quantitative maps were obtained using software processing. The quantitative maps and structural plots of both groups were compared and analyzed, expecting to gain specific evidence for MRI diagnosis of ADHD-ATs children.

## Materials and methods

### Ethics statement

This study was approved by the Ethics Committee of the Children's Hospital of Chongqing Medical University (No. 2,019–221), and the family members signed informed consent forms prior to the study.

### Patient information

#### Study group

A total of 56 children with ADHD-Ats, who were aged 4–5 years, were prospectively selected from March 2020 to June 2021, of which 38 children were sedated.

#### Control group

A total of 53 children with ADHD without ATs, who were aged 4–5 years, were prospectively selected from May 2020 to July 2021, of which 36 children were sedated. There was no marked differences in age, sex ratio, body weight, BMI value, trace elements or iron detection values between the research group and the control group (*p* > 0.05) (Table 1).

**Table 1:** Patient information.

** 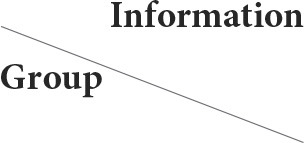 **	**Male to female ratio**	**Age (years)**	**Weight (kg)**	**BMI**	**Trace iron (umol/L)**	**Hemoglobin (g/L)**	**MCV (fL)**	**Sedation patients**	**Scan times**
ADHD (without ATs)	26:27	4.49 ± 0.17	18.27 ± 2.12	16.27 ± 1.35	8.23 ± 1.87	135.87 ± 21.69	87.63 ± 5.87	36	18.25
ADHD-ATs	29:27	4.51 ± 0.16	18.32 ± 1.87	16.33 ± 156	8.31 ± 1.98	137.76 ± 23.45	85.98 ± 4.92	38	18.13
X^2^/T-value	0.081	−0.633	−0.131	−0.003	−0.217	−0.436	1.594	0.000	0.000
*P*-value	0.776	0.528	0.896	0.998	0.829	0.664	0.114	0.994	0.996

#### Inclusion criteria

The body mass index of children ranged from 14 to 16, they were right-handed, and they met the diagnostic criteria stipulated in the Diagnostic and Statistical Manual of Mental Disorders (DSM–V) for ADHD or ADHD-ATs (27). The children reported no additional nervous system functional diseases, no concomitant diseases of other organs, no additional diseases affecting brain function and structure, no history of drug treatment, and serum iron levels within the normal range (6.5–9.85 μmol/L). Routine head MRI examinations were normal.

The children with ADHD or ADHD-ATs included in this study all had preliminary diagnoses at the outpatient clinic by a clinician with a deputy senior title or above in the Department of Child Development and Behavior, Child Health Care and Department of Psychology in our hospital. The children included met the diagnostic criteria for ADHD or ADHD-ATS in the Diagnostic and Statistical Manual of Mental Disorders, Fifth Edition.

All children who did not cooperate with the examination accepted the services of the Sedation Center of our hospital before examination. The sedation methods included a nasal drip of dexmedetomidine 3 ug/kg + oral administration of chloral hydrate 40 mg/kg. After 20 min, if the sedation was not satisfactory, a nasal drip of dexmedetomidine 1 μg/kg was given again, and oral administration of chloral hydrate 20 mg/kg was given if necessary.

### Equipment and methods

A discovery MR750 3.0T magnetic resonance scanner (GE Medical Systems, Milwaukee, WI, USA) was used and included an 8-channel head and neck joint coil. All the children who did not cooperate accepted the services of the Sedation Center and fell asleep prior to receiving 3D-T1, ESWAN, 3D-PCASL, T1 FLAIR, T2 FLAIR, and T2 WI sequence scanning. ESWAN: FOV, 24 cm; slice thickness, 1 mm; TR, 81.8 ms; flip angle, 20; TE, 4 ms; locs per slab, 150; slab, 1; scanning time, 7 min and 47 s. 3D-T1: FOV, 24 cm; slice thickness, 1 mm; TR, 7.9 ms; flip angle, 12; TE, 3.1 ms; 156 layers; scanning time, 3 min and 43 s. 3D-PCASL: FOV, 24 cm; slice thickness, 4 mm; TR, 4,628 ms; PLD, 1,525 ms; slab, 1; 36 layers; scanning time, 4 min and 29 s.

#### Data analysis

**Figure 1:**
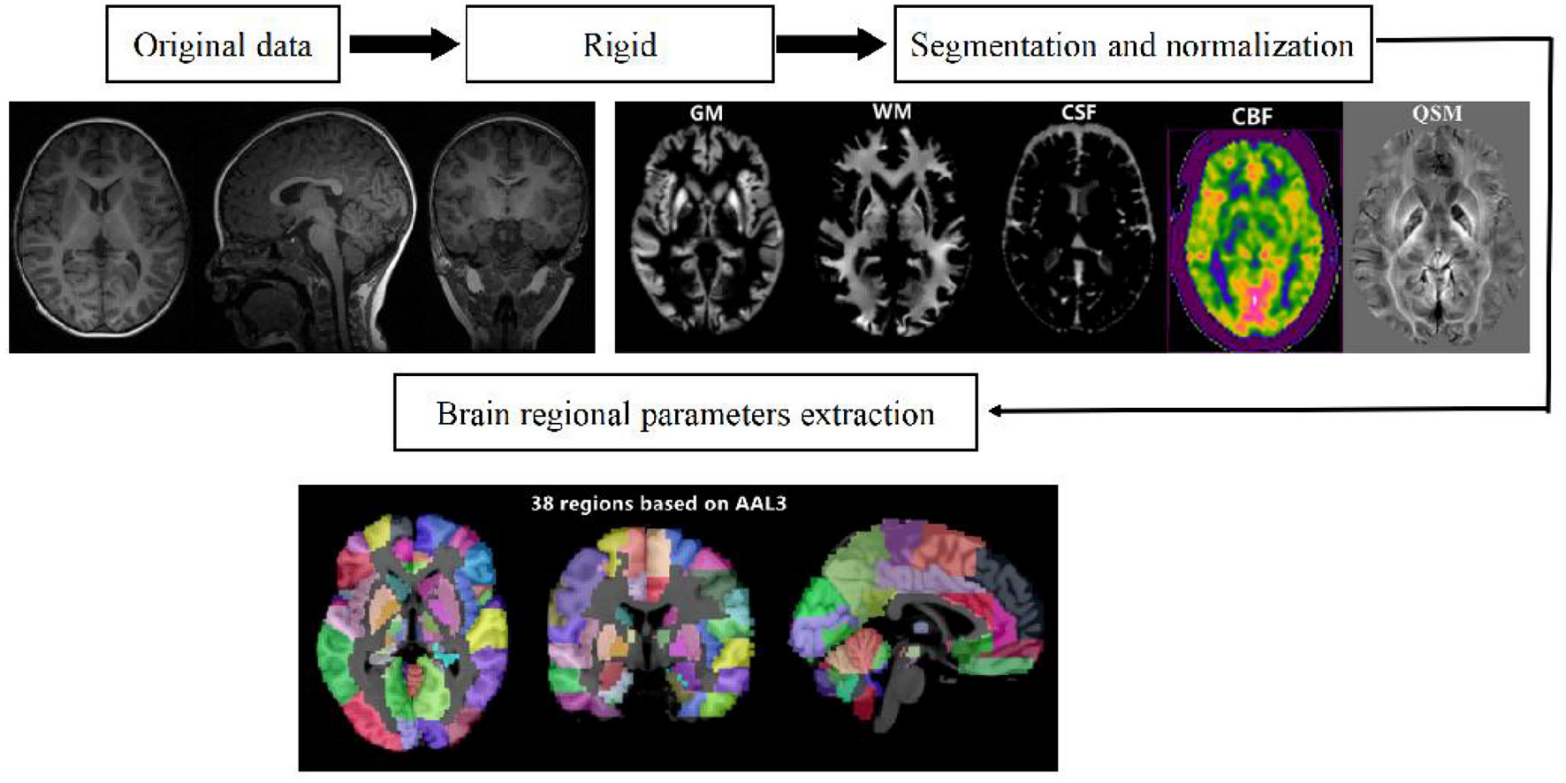
Schematic diagram of the image processing and parameter extraction. Shilong Tang, Guanping Zhang, Qiying Ran, Xianfan Liu, Zhengxia Pan, Ling He. Quantitative susceptibility mapping shows lower brain iron content in children with attention-deficit hyperactivity disorder. Human brain mapping/equipment and method, pages no.2497, copyright (2022), with permission from. Reprinted from human brain mapping. 2022, 43(8):2495–2502.

Raw 3D-PCASL data for the CBF quantitative map were acquired *via* the GE ADW4.6 workstation, and the raw ESWAN data for the QSM quantitative map were acquired through the MATLAB 2018a (MathWorks, Natick, MA, USA) platform using STI Suite 3.0 software (https://people.eecs.berkeley.edu/chunlei.liu/software.html). To calculate the quantitative parameters CBF and QSM in different brain regions, we adopted a voxel-based morphometry (VBM) method and employed SPM12 software *via* the MATLAB 2018a platform (http://www.fil.Ion.ucl.ac.uk/spm/); the 3D-T1 sequence structure map was registered with quantitative CBF and QSM maps. Subsequently, the CAT12 toolkit in SPM12 software (http://www.neuro.uni-jena.de/cat/) was employed to segment the registered structural quantitative map and ultimately extract the parameter values for each brain area (Figure 1).

### Statistical analysis

SPSS 25.0 statistical software was adopted, and the measurement data were expressed as $\bar{X}$ ± s. The chi-square test was performed to compare the sex ratio of the children in the research group and the control group. *T*-tests with the two independent samples were employed to compare the age, weight, BMI value, and iron values using trace element detection of the children between the research group and the control group. The iron content, CBF value, and brain volume of the children in the research group and the control group were compared using two independent sample *t*-tests (*p* < 0.05 was considered statistically significant). Receiver operating characteristic (ROC) curve analysis was adopted to evaluate iron content values and CBF values in particular brain regions and for ADHD-ATs diagnostic value. When the AUC was >0.5 and there was statistical significance, the parameters were considered to have diagnostic value; the closer to 1 the value was, the higher the diagnostic value.

## Results

### Comparison results of the trace element iron, hemoglobin, and red blood cell volume in blood

There were no significant differences in blood levels of the trace element iron and hemoglobin and red blood cell volume and sedation number between the study group and the control group (*p* > 0.05) (Table 1).

### Comparison of iron content in different brain regions

Iron content in the frontal lobe, temporal lobe, hippocampus, and caudate nucleus of the children with ADHD-ATs were lower than those in the children with ADHD without ATs (*p* < 0.05). The total iron content in the brains of children with ADHD without ATs was higher than that in children with ADHD-ATs (*p* < 0.05) (Table 2).

**Table 2:** Comparison of the iron content/CBF value between the ADHD-ATs and ADHD (without ATs) groups.

** 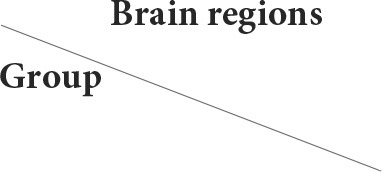 **	**Frontal**	**Hippocampus**	**CN**	**PU**	**GP**	**TH**	**Temporal**	**SN**	**RN**
**Iron content [**$\bar{x}$ **±s, ppb (× 10**^**−9**^**)]**									
ADHD (without ATs)	133.52 ± 24.16	145.62 ± 26.33	725.32 ± 143.12	60.45 ± 13.28	974.52 ± 165.33	362.58 ± 70.54	193.46 ± 49.52	462.41 ± 97.12	215.52 ± 46.17
ADHD-ATs	120.26 ± 22.13	131.38 ± 19.04	643.25 ± 121.27	59.02 ± 10.57	963.19 ± 114.25	355.42 ± 81.17	176.85 ± 31.30	458.23 ± 69.94	213.10 ± 42.01
*T*-value	2.990	3.248	3.236	0.624	0.418	0.490	2.105	0.259	0.286
*P*-value	0.003	0.002	0.002	0.534	0.677	0.625	0.038	0.796	0.775
**CBF (mL/100 g·min)**									
ADHD (without ATs)	69.33 ± 5.78	58.34 ± 8.76	55.62 ± 6.15	66.52 ± 5.71	63.34 ± 7.83	52.23 ± 6.98	76.34 ± 7.25	60.12 ± 8.57	68.23 ± 7.53
ADHD-ATs	63.24 ± 5.61	49.46 ± 9.23	48.14 ± 9.13	57.18 ± 4.89	51.29 ± 6.97	50.15 ± 7.43	69.23 ± 6.13	58.62 ± 8.21	66.01 ± 8.49
*T-*value	5.582	5.146	4.988	9.188	8.497	1.504	5.539	0.933	1.441
*P*-value	< 0.001	< 0.001	< 0.001	< 0.001	< 0.001	0.135	< 0.001	0.353	0.152

TH, Thalamus; GP, Globus pallidus; SN, Substantia nigra; RN, Red nucleus; CN, Caudate nucleus; PU, Putamen.

### Comparison of CBF values in different brain regions

The CBF values in the frontal lobe, temporal lobe, globus pallidus, caudate nucleus, putamen, and hippocampus of children with ADHD-ATs were lower than those of children with ADHD without ATs (*p* < 0.05). The total brain CBF value for children with ADHD-ATs was lower than that of children with ADHD without ATs (*p* < 0.05) (Table 2).

### Brain area volume comparison

There were no significant differences in the volume of each brain area of the children in the study group and the control group (*p* < 0.05) (Table 3).

**Table 3:** Volume values of brain regions in children [$\bar{x}$ ± s, volume (cm^3^)].

** 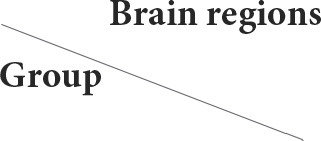 **	**ADHD (without Ats)**	**ADHD-ATs**	***T*** **-value**	***P*** **-value**
Frontal	119.25 ± 12.24	118.24 ± 16.87	0.356	0.722
Temporal	123.45 ± 20.38	120.59 ± 25.13	0.650	0.517
Hippocampus	8.29 ± 1.56	8.31 ± 1.64	−0.065	0.948
TH	9.81 ± 2.35	9.75 ± 2.62	0.126	0.900
GP	1.56 ± 0.33	1.65 ± 0.38	−1.317	0.191
SN	0.17 ± 0.05	0.16 ± 0.05	1.044	0.299
PU	13.02 ± 3.28	12.99 ± 3.14	0.049	0.961
RN	0.0059 ± 0.0005	0.0058 ± 0.0004	1.156	0.250
CN	6.89 ± 0.93	6.95 ± 1.03	−0.319	0.751
Whole brain	1,389.43 ± 87.92	1,378.55 ± 88.63	0.643	0.522

TH, Thalamus; GP, Globus pallidus; SN, Substantia nigra; RN, Red nucleus; CN, Caudate nucleus; PU, Putamen.

### ROC analysis

**Figure 2:**
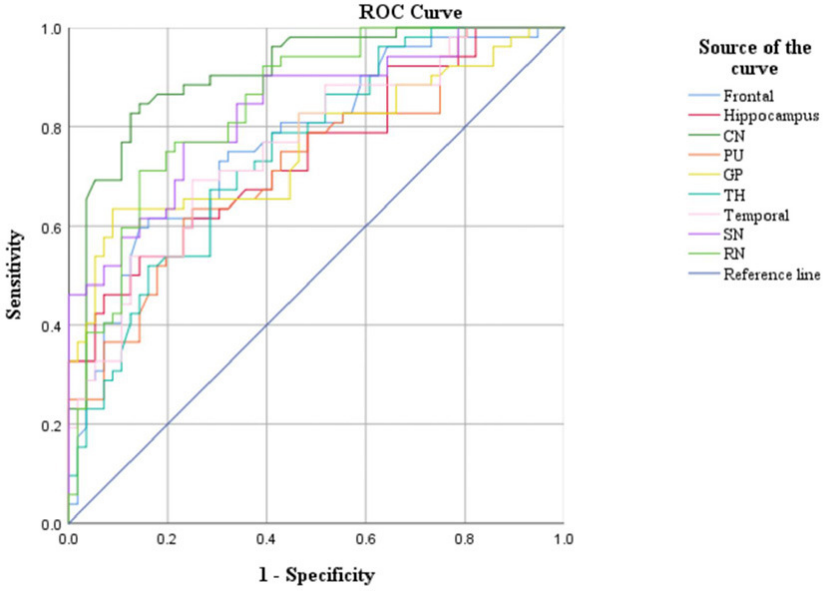
ROC curve analysis results of iron content in brain regions.

**Figure 3:**
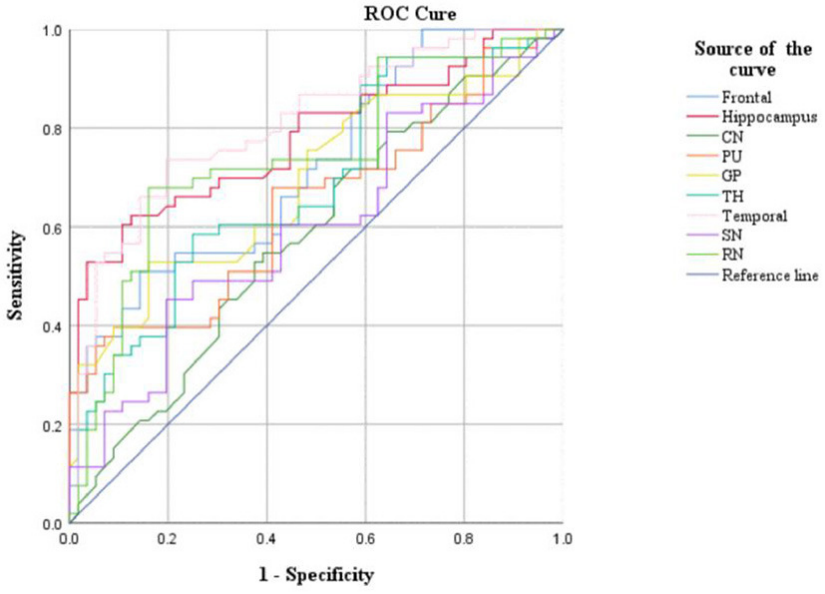
ROC curve analysis results of CBF in brain regions.

CBF and QSM values in the frontal lobe, temporal lobe, and caudate nucleus could distinguish children with ADHD-ATS from children with ADHD without ATs (AUC > 0.05, *p* < 0.05); the AUC value obtained *via* the data obtained with the QSM method was the highest (0.907) (Figures 2, 3; Table 4).

**Table 4:** ROC curve analysis results of iron content/CBF in brain regions of ADHD-ATs children (*n* = 102).

**Brain region**	**AUC**	**Std. Error**	***P*** **-value**	**95% CI**	
				**Lower bound**	**Upper bound**
**Iron content**					
Frontal	0.772	0.045	0.000	0.683	0.860
Hippocampus	0.743	0.047	0.000	0.650	0.835
CN	0.907	0.029	0.000	0.851	0.963
PU	0.723	0.049	0.065	0.628	0.819
GP	0.764	0.047	0.138	0.672	0.857
TH	0.751	0.046	0.256	0.661	0.841
Temporal	0.760	0.046	0.098	0.671	0.850
SN	0.832	0.039	0.075	0.756	0.908
RN	0.844	0.037	0.084	0.771	0.917
**CBF**					
Frontal	0.719	0.048	0.000	0.625	0.814
Hippocampus	0.780	0.045	0.000	0.692	0.868
CN	0.580	0.055	0.048	0.473	0.688
PU	0.643	0.053	0.070	0.538	0.748
GP	0.691	0.051	0.501	0.591	0.791
TH	0.685	0.051	0.892	0.585	0.784
Temporal	0.810	0.041	0.654	0.729	0.891
SN	0.600	0.055	0.071	0.493	0.708
RN	0.739	0.049	0.096	0.644	0.835

TH, Thalamus; GP, Globus pallidus; SN, Substantia nigra; RN, Red nucleus; CN, Caudate nucleus; PU, Putamen.

## Discussion

The 3D-PCASL technique uses water molecules in blood in the arteries as a contrast agent for vascular perfusion imaging. Compared with the conventional perfusion imaging using an intravenous bolus injection of contrast agents, the greatest strength of 3D-PCASL vascular perfusion imaging is its non-invasiveness, which requires no contrast agent injection, and the blood perfusion value of the corresponding part of the client can be repeatedly detected (28–31).

Quantitative susceptibility mapping (QSM) is a relatively new technique developed on the basis of SWI imaging technology in recent years. Compared with conventional SWI, which is based on magnetic susceptibility difference imaging, the application of the QSM technique to quantitatively determine brain iron content enables the detection of minor changes in iron content in the brain and provides better specificity in the spatial image contrast, thereby improving sensitivity and reliability (32–37).

The results of the study revealed that both the cerebral blood flow and brain iron content in the frontal lobe, temporal lobe, hippocampus, and caudate nucleus of the children in the study group were lower than those in the control group. The iron content decrease in the brain regions of the children in the research group might have been caused by decreased brain blood flow. Iron is mainly present as heme- and non-heme iron in the human body. Heme iron mainly exists in the form of oxyhemoglobin, deoxyhemoglobin, and methemoglobin in the blood, accounting for ~2/3 of the total iron content in the human body. Non-heme iron is present in the blood as ferritin and hemosiderin, and over 1/3 of non-heme iron present in the brain is in the form of ferritin. As cerebral blood flow decreases, both heme iron and non-heme iron, which is in the blood, are reduced, that eventually leads to a decrease in the iron content in these brain areas (37, 38). In the study group, the partial decrease in cerebral blood flow did not cause a decrease in iron content. It might be that the decrease in cerebral blood flow in this part of the brain region was not marked, resulting in no significant decrease in brain iron content.

The results indicated that there were no significant differences in the measures of trace element iron, hemoglobin, and red blood cell volume between the children in the research group and the healthy children. However, the QSM technique was sensitive to reduced iron content in some brain regions in the autistic children, indicating that iron content in several brain regions might start to decline even when there was no significant change in the detection of trace element iron in the blood of autistic children at an early stage. The QSM technique was more sensitive for monitoring changes in iron content in some brain regions in the autistic children. The research results also suggested that there was no significant difference in brain volumes between the children in the study group and the control group, whereas the brain iron content and cerebral blood flow parameter values in some brain regions in the children in the research group were abnormal, indicating that such abnormalities might not be correlated with the volume of the brain regions.

Moreover, CBF and QSM techniques in the frontal lobe, temporal lobe, and caudate nucleus brain regions could distinguish the children with ADHD-ATs (AUC > 0.5, *p* < 0.05), and the QSM technique presented with higher AUC values. Therefore, the three brain regions could be focused on as key regions for brain imaging in the diagnosis of children with ADHD-ATs, and the QSM technique could be applied as the optimal diagnostic option.

The frontal lobe is the most developed brain lobe in the human body, with multiple key functions. The first function is thinking, including calculating and logical thinking, which is mostly completed in the frontal lobe. The second function is verbal capability, especially verbal expression, language organization, and language fluency, which is determined by the frontal lobe (39, 40). Additionally, the motor center is located in the frontal lobe, and the formation of personality and behavior patterns can be affected if the frontal lobe is underdeveloped (41, 42). The study findings revealed that the iron content and cerebral blood flow in the frontal lobe of the children with ADHD-ATs were lower than those of the children with ADHD without ATs, which led to poorer frontal lobe development and thereby caused poorer development of language, thoughts, and mobility in the children with ADHD-ATs than in the children with ADHD without ATs.

The main functions of the temporal lobe are linked to the auditory sense, language comprehension, memory and mental activity. The posterior part of the superior temporal gyrus in the cerebral hemisphere belongs to the sensory language center, and sensory aphasia occurs once it is injured. These manifestations include patients hearing their voice when speaking to oneself, but the patient is unable to understand the meaning of other individuals or their own words. Furthermore, the patient fails to understand the questions of others (43, 44). The study findings revealed that iron content and cerebral blood flow in the temporal lobe of the children with ADHD-ATs were lower than those of the children with ADHD without ATs, which led to poorer temporal lobe development and thereby caused poorer development of understanding in the children with ADHD-ATs than the children with ADHD without ATs.

The caudate nucleus acts as an essential part of the learning and memory system of the brain. It not only regulates body movement but also affects the sensory conduction process to a certain degree. Anatomical data have also confirmed direct projection fibers from the head of the caudate nucleus to the neocortex, cingulate gyrus, and brainstem. Stimulation of the caudate nucleus can increase pain thresholds, while damage to the caudate nucleus can impair this effect. Therefore, the caudate nucleus may exert an essential role in the regulatory mechanism of pain transmission (45, 46). The study findings revealed that iron content and cerebral blood flow in the caudate nucleus of the children with ADHD-ATs were lower than those of the children with ADHD without ATs, which led to poorer caudate nucleus development and thereby caused poorer development of learning and memory capabilities in the children with ADHD-ATs than the children with ADHD without ATs.

In the study, we used the ALL-3 template. In the previous preliminary study, we compared the data values of brain regions obtained by software with the data values of brain regions obtained by manually delineating the ROI of the same group of children, and found that the results were consistent. Therefore, the data obtained in this study were highly accurate.

In the study, we compared the number of test group children with that in the control group, and the results showed that there was no statistically significant difference (*P* = 0.994). We also made statistical analysis on the index such as age, weight, BMI, etc., but the difference was not statistically significant (*P* > 0.05). There was also no difference in the dosage of sedatives used by the children in the two groups (the dosage of sedatives was determined according to weight and BMI). Therefore, Sedation did not lead to bias in the final study results.

In the study, we compared the gender of two groups, the results showed that there was no statistically significant difference (*P* = 0.776). For that reason, we did not pay further attention to the impact of gender on the results.

This study also has some limitations. Only children aged 4–5 years were enrolled in this study, and children beyond this age range were not included. The present research was a single-center study and limited to a certain region. The study used sedation for some children who did not cooperate, which might have impacted the results of the study [e.g., cerebral CBF values in children might be reduced after sedation (47, 48)]. The abovementioned limitations need to be further improved in future research.

Taken together, iron content and cerebral blood flow in the frontal lobe, temporal lobe, and caudate nucleus of children with ADHD-ATs were lower than those of children with ADHD without ATs, and quantitative magnetic resonance imaging (QSM and ASL) could distinguish children with ADHD-ATs from those with ADHD without ATs. The QSM technique might be the optimal diagnostic technique for children with ADHD-ATs.

## Data availability statement

The original contributions presented in the study are included in the article/supplementary materials, further inquiries can be directed to the corresponding author/s.

## Ethics statement

This study was approved by the Ethics Committee of the Children's Hospital of Chongqing Medical University (No. 2,019–221), and the family members signed informed consent forms prior to the study. Written informed consent to participate in this study was provided by the participants' legal guardian/next of kin.

## Author contributions

ST and LH contributed to experimental design and project management. XL and ZC contributed to data acquisition and data analysis. QR contributed to data statistics. LN provided software support. All authors contributed to the article and approved the submitted version.

## Funding

This study protocol was supported by grants from National Clinical Research Center for Child Health and Disorders (Children's Hospital of Chongqing Medical University, Chongqing, China) (grant number NCRCCHD-2021-YP-07) and Chongqing Municipal Education Commission (No. KJQN202000425).

## Conflict of interest

Author LN was employed by the company GE Healthcare. The remaining authors declare that the research was conducted in the absence of any commercial or financial relationships that could be construed as a potential conflict of interest.

## Publisher's note

All claims expressed in this article are solely those of the authors and do not necessarily represent those of their affiliated organizations, or those of the publisher, the editors and the reviewers. Any product that may be evaluated in this article, or claim that may be made by its manufacturer, is not guaranteed or endorsed by the publisher.

## Abbreviations

ADHD: Attention-deficit hyperactivity disorder
MRI: magnetic resonance imaging
ESWAN: enhanced T2^*^-weighted angiography
ROI: region of interest
DSM-V: Diagnostic and Statistical Manual of Mental Disorders Fifth Edition
QSM: quantitative susceptibility mapping
MCV: mean corpuscular volume
CSF: cerebrospinal fluid
SWI: susceptibility weighted imaging
MCV: mean corpuscular volume
VBM: voxel-based morphology
CBF: cerebral blood flow
PLD: post-labeling delay
ATs: autistic traits
3D-PCASL: three-dimensional pseudocontinuous arterial spin labeling.
